# Towards Standards for Human Fecal Sample Preparation in Targeted and Untargeted LC-HRMS Studies

**DOI:** 10.3390/metabo11060364

**Published:** 2021-06-07

**Authors:** Farideh Hosseinkhani, Anne-Charlotte Dubbelman, Naama Karu, Amy C. Harms, Thomas Hankemeier

**Affiliations:** Leiden Academic Center for Drug Research, Division of System Biomedicine and Pharmacology, Leiden University, 2300 RA Leiden, The Netherlands; f.hosseinkhani@lacdr.leidenuniv.nl (F.H.); a.c.dubbelman@lacdr.leidenuniv.nl (A.-C.D.); n.karu@lacdr.leidenuniv.nl (N.K.)

**Keywords:** fecal metabolites, gut microbiota, metabolomics, sample preparation, LCMS (liquid chromatography mass spectrometry)

## Abstract

Gut microbiota and their metabolic products are increasingly being recognized as important modulators of human health. The fecal metabolome provides a functional readout of the interactions between human metabolism and the gut microbiota in health and disease. Due to the high complexity of the fecal matrix, sample preparation often introduces technical variation, which must be minimized to accurately detect and quantify gut bacterial metabolites. Here, we tested six different representative extraction methods (single-phase and liquid–liquid extractions) and compared differences due to fecal amount, extraction solvent type and solvent pH. Our results indicate that a minimum fecal (wet) amount of 0.50 g is needed to accurately represent the complex texture of feces. The MTBE method (MTBE/methanol/water, 3.6/2.8/3.5, *v*/*v*/*v*) outperformed the other extraction methods, reflected by the highest extraction efficiency for 11 different classes of compounds, the highest number of extracted features (97% of the total identified features in different extracts), repeatability (CV < 35%) and extraction recovery (≥70%). Importantly, optimization of the solvent volume of each step to the initial dried fecal material (µL/mg feces) offers a major step towards standardization, which enables confident assessment of the contributions of gut bacterial metabolites to human health.

## 1. Introduction

Gut microbial metabolites are rapidly becoming a key facet of human health and disease [1,2,3]. In this context, the fecal sample has emerged as the most accessible biological matrix, which reflects the functional readout of microbial activity in the gastrointestinal tract and probes the reciprocal regulation between gut microbiota and the physiology of the host [4]. Several previous studies have proposed the versatility of fecal metabolites in disease diagnosis. For example, elevated concentrations of amino acids, saturated fatty acids, and ursodeoxycholic acid were reported in fecal samples of colorectal cancer patients [5], whereas increased levels of deoxycholic acid, L-tryptophan and putrescine were found in fecal samples of patients with systemic lupus erythematosus [6]. Short chain fatty acids, polyphenols and vitamins have been shown to decrease the risk of inflammatory bowel disease, cancer, diabetes and cardiovascular diseases [7,8]. Additional correlations will likely emerge soon. Thus, examining the fecal metabolome serves as a robust approach for understanding the interaction between human metabolism and the gut microbiota composition in health and disease.

Despite the rising popularity of fecal metabolomics, the methods for fecal sample preparation and analysis are still a long way from being standardized [9,10]. In recent years, various studies on human fecal metabolomics aimed to provide guidelines for fecal sample collection, storage and sample pre-treatment. Sample storage at −80 °C has been introduced as the ‘’golden standard’’ for both microbiome and metabolomics studies, as the use of frozen samples in human studies is both more common and practical, while minimizing fecal microbial changes and metabolite variation [11,12,13,14]. For a uniform sample handling procedure in metabolomics studies, homogenization and aliquoting of the fecal sample prior to freezing has been suggested by Karu et al. [9].

However, controversy remains between studies regarding the critical parameters of fecal extraction such as appropriate sample size, extraction solvent type and solvent pH. One of the main challenges in fecal metabolomics is the high heterogeneity and complexity of fecal samples, which limits repeatability and efficiency of metabolite extraction. In addition, the fecal metabolome contains a wide range of metabolites with diverse structures and different chemical properties [15]. The selection of the extraction solvent can strongly bias the outcome of the study as it significantly affects the loss, underestimation or overestimation of the metabolites’ recovery. To obtain a thorough metabolite extraction that is analyzable with good coverage, efficiency and repeatability, compromises in the sample (pre)treatment have to be made. The lack of a well-defined and standardized protocol causes independent studies to report different metabolite coverages and distinct quantities of metabolite biomarkers, due to the utilization of different sample treatment platforms, which impedes biological interpretation [16]. This illustrates the importance of data generation and reporting in a unified manner, and highlights the need to standardize sample preparation and analysis workflow.

In recent years, liquid chromatography mass spectrometry (LCMS) has emerged as the most commonly used technique for global metabolic profiling, due to its sensitivity, and capability for resolving various classes of metabolites [17]. Therefore, in this study, we aimed at designing a standardized human fecal sample preparation methodology for targeted and untargeted metabolomic analysis using high resolution LCMS. We evaluated in particular the quantity of starting fecal material for extraction, the effect of solvent type and pH on the extraction efficiency, and the effect of the extraction methods on the extraction coverage, repeatability and recovery.

## 2. Results

### 2.1. Sample Preparation Methods and Workflow

**Figure 1:**
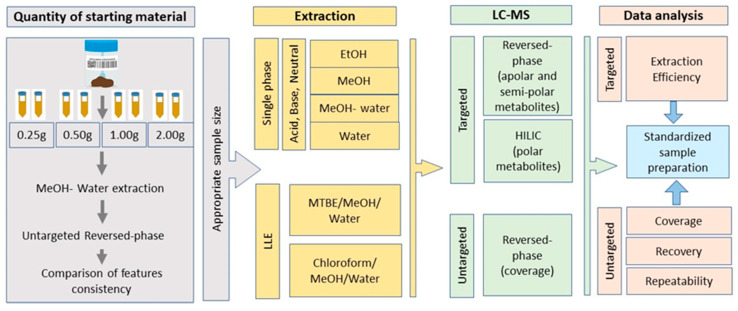
Experimental workflow and sample treatment evaluation.

**Figure 2:**
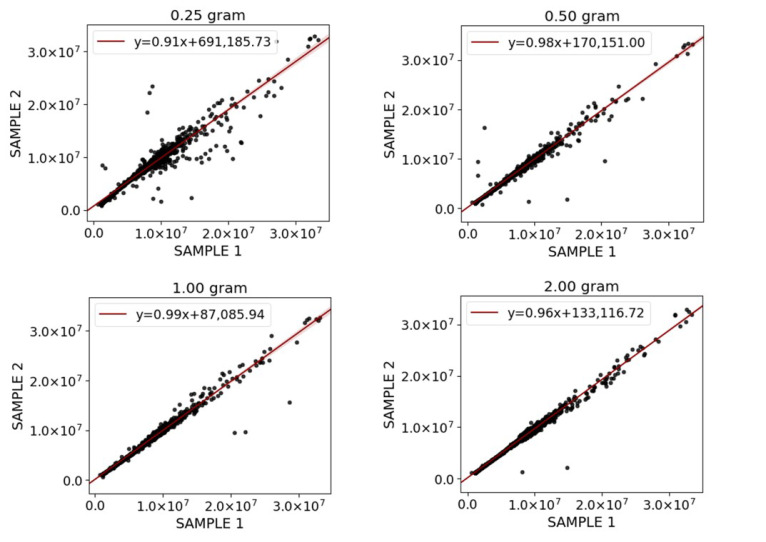
Effect of fecal amount (0.25, 0.50, 1.00 and 2.00 g) on feature intensities of two aliquots taken from the same homogenized fecal scoop. Black dots represent intensities of each feature. A linear relation between feature intensities of two subsamples from the same fecal scoop indicates a consistent representation of the sample.

Fecal samples from healthy volunteers (*n* = 3) were collected for this study. The fresh samples were homogenized by stirring, aliquoted and stored directly at −80 °C until extraction. The experimental workflow is illustrated in Figure 1. First, the effect of starting fecal amount on the extraction repeatability was assessed for four different sample sizes (in duplicate) using untargeted reversed-phase LC-MS (RPLC-MS); see method 1 in Table S2. This experiment was performed on aliquots from an individual fecal sample to exclude bias due to individual sample differences (Figure 2).

**Figure 3:**
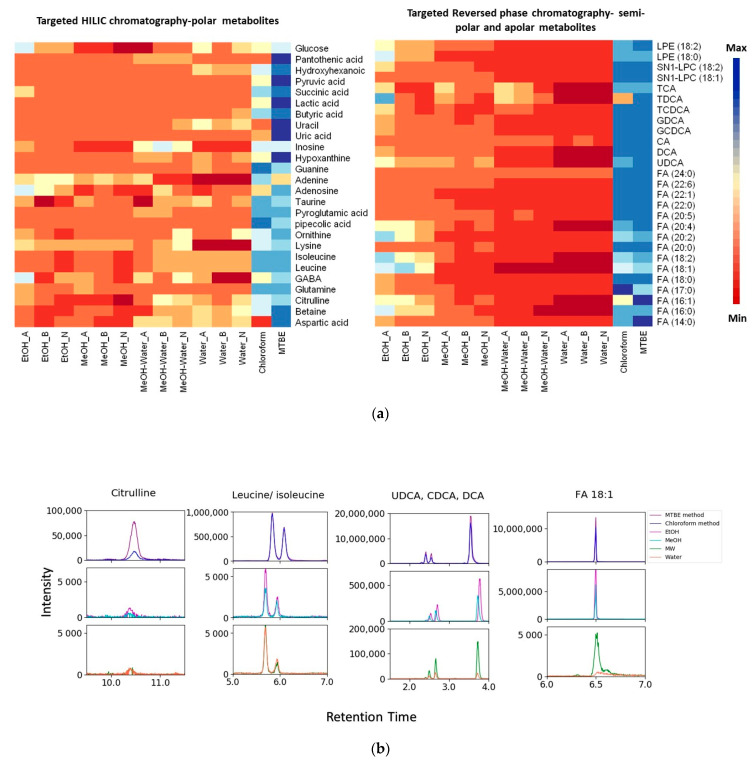
The effect of the extraction solvent system on the extraction efficiency. (**a**) Heatmap of the peak area of the metabolites from different classes of compounds using single-phase (first cycle of extractions with different solvents and pH values) and two-phase liquid–liquid extraction (MTBE and chloroform methods). Dark blue and dark red colors represent the largest and smallest peak area, respectively. (**b**) Peak shapes and intensity of metabolites extracted with different extraction solvents in neutral pH. -A: acidic, -B: basic, -N: neutral. FA: fatty acid; UDCA: ursodeoxycholic acid; CDCA: chenodeoxycholic acid; DCA: deoxycholic acid.

Next, side-by-side comparisons of four single-phase extractions (EtOH, MeOH, MeOH-water and water) in three different pH (acidic, neutral and basic), and two liquid–liquid extractions (MTBE and chloroform methods) were performed to evaluate the efficiency, coverage, recovery and repeatability of extraction of polar and apolar metabolites from fecal samples. For this experiment, samples from the three volunteers were pooled (see Materials and Methods Section 4.4). For extraction efficiency, the extracts were analyzed in a targeted manner using RPLC-MS (method 2 in Table S2) and hydrophilic interaction liquid chromatography (HILIC)-MS (method 3 in Table S2). Both platforms were operated only in negative mode (see Section 4). The choice of ionization mode was based on our laboratory R&D process and previously published data [18,19], which showed that most polar compounds are ionized better in negative ion mode. Compounds such as acylcarnitines, which ionize only in positive ESI, are not included in our target list. Information on the performance of the targeted methods used can be found in Supplementary Material Tables S3 and S4 (excel files). These techniques targeted a total of 27 semi- and apolar compounds from three different classes (fatty acyls, steroids and steroids derivatives and glycerophospholipids), and 26 polar compounds from eight different classes (organooxygen compounds, hydroxy acids and derivatives, keto acids and derivatives, carboxylic acids and derivatives, diazines, purine nucleotides, imidazopyrimidines and organic sulfonic acids and derivatives). The target list was selected to include representative fecal metabolites from classes important for characterizing gut microbiota, diet and human health [3,20,21,22,23]. The classification of metabolites was based on the Human Metabolome Database (HMDB) [24]. The relative abundance of each detected metabolite (only the first extract) was used to evaluate the extraction efficiency for each corresponding metabolite class (Figure 3).

**Figure 4:**
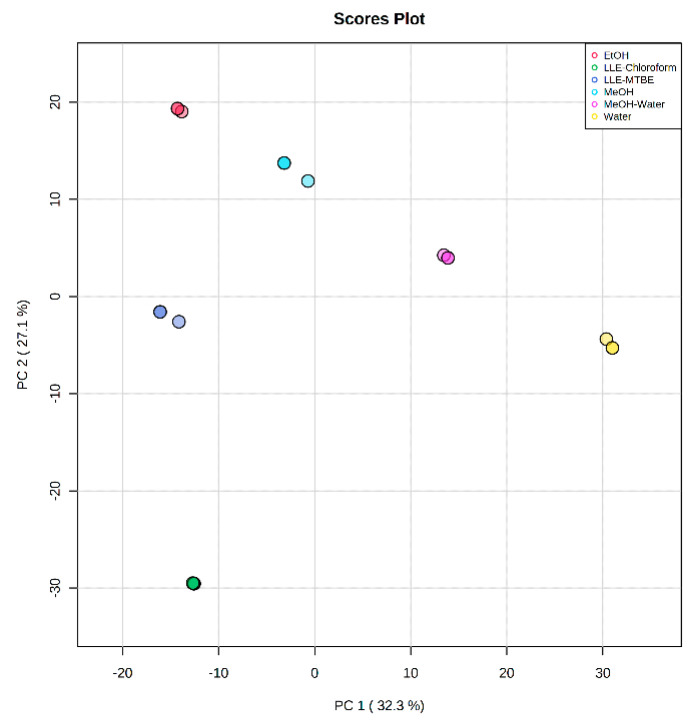
Principle component analysis (PCA) scores plot of pre-processed untargeted features (*n* = 2176) measured in the different fecal extraction procedures (*n* = 3 replicates).

For extraction coverage, extracts were analyzed using untargeted RPLC-MS (method 1) and the total number of features from each extract was determined using an XCMS pipeline (Figure 4, Tables S5 and S6). Finally, extraction repeatability and recovery were examined for extraction methods with higher feature coverage using isotopically labeled standards and untargeted RPLCMS (method 1).

### 2.2. Quantity of Starting Material

In this study, two aliquots of each size (0.25, 0.50, 1.00 and 2.00 g) were taken from the same homogenized fecal scoop. Each sample was measured in triplicate and the mean intensities of features from each sample size was compared and plotted against the mean intensity of features from the corresponding sample size (Figure 2). We observed that a minimum of 0.50 g wet feces from the whole scoop was needed as starting material, and smaller sample sizes (≤0.50 g wet feces prior to freeze drying) tended to suffer from a relatively higher bias. Although samples of 1.00 and 2.00 g exhibited the highest coefficient of determination (R^2^ = 0.9804 and 0.9863, respectively), practical difficulties arose regarding the volume of solvent needed for extraction; in particular when the sample was treated with LLE. In this study, a sample size of 0.50 g was chosen for further steps as it showed negligible variance for replicate analysis (R^2^ = 0.9629).

### 2.3. Assessment of Extraction Method, Solvent and pH for Optimal Extraction Efficiency

The extraction efficiency was affected by the type of solvent, polarity and pH value of extraction solvents. Owing to the diverse classes of compounds in our target list (53 compounds from 11 different biochemical classes) the obtained results can be extrapolated to vast numbers of metabolites with diverse chemical properties.

#### 2.3.1. pH Effect of Extraction Solvent

The effect of solvent pH on extraction efficiency was assessed by comparing three different pH values (acidic, basic and neutral) for solvents in single-phase extractions. Peak areas of 53 analytes of interest (measured in triplicate) were plotted in a heatmap (Figure 3a). Basic pH, independent of the solvent used, tended to provide the lowest efficiency across the wide range of metabolites. The extraction yield of fatty acyls, steroids and steroids derivatives, and glycerophospholipids was higher with acidic EtOH, while carboxylic acids such as ornithine, lysine and leucine had a higher yield in neutral pH; purine nucleotides and imidazopyrimidines showed a higher yield with basic pH. Due to the different charges of metabolites, we could not find one ideal pH value that was optimal for the full range of metabolites in our experiment.

#### 2.3.2. Effect of Solvent Selection

The appropriate solvent selection for single-phase extractions is a subject of debate. Similar to the results obtained for pH, as expected, there is no ideal solvent for single-phase extractions with high efficiency for all types of metabolites (Figure 3a, columns with EtOH, MeOH, MeOH-water (MW) and water extraction under three different pH values). Specifically, a higher yield for fatty acids (fatty acyls), LPE, LPC (glycerophospholipids), bile acids (steroids and steroids derivatives), glucose (organooxygen compounds) and adenosine (purine nucleoside) was observed with acidified EtOH, whereas some carboxylic acids and derivatives (ornithine, lysine and GABA) showed a better extraction yield with MeOH-water. Others such as taurine, betaine, leucine and isoleucine were better extracted with water.

#### 2.3.3. LLE Solvent Effect

To circumnavigate the chemical limitations and coverage of single solvent extractions, we performed two different LLE methods using MTBE and chloroform. Slight modifications were implemented to obtain a better solvent ratio of MTBE/MeOH/water (3.6/2.8/3.5, *v*/*v*/*v*) for extraction of both polar and apolar metabolites (see Materials and Methods Section). Although, the total volume of solvents in LLE is 10.2 μL, the volume of polar solvent in LLE (MeOH and water) is similar to those in single-phase extractions. We considered a very low to no effect of MTBE/ chloroform on the extraction of polar metabolites. Therefore, to ensure a fair comparison, the volume collected from each layer of LLE to be dried and reconstituted was the same as the volume collected from single-phase extractions. Aqueous and organic layers of LLE were analyzed separately for assessing the extraction efficiency for polar and apolar metabolites. For a fair analysis, we first compared the efficiency based on the first cycle of single-phase extractions. In comparison to single-phase extractions, LLE with either of the methods showed a higher or comparable extraction efficiency (Figure 3a, columns entitled MTBE and chloroform). However, the MTBE method outperformed the chloroform method for polar metabolites, in terms of superior peak shapes and intensities (Figure 3b). In particular, the MTBE method demonstrated a higher yield of organic acids, such as pyruvic acid, succinic acid and lactic acid. Moreover, single-phase extractions such as with EtOH require at least three cycles of extraction, as well as solution pH optimization (dependent on the class of compounds) to obtain a comparable efficiency with LLE (Supplementary Figure S1).

#### 2.3.4. Metabolic Coverage

Untargeted metabolomics analysis of each extraction method was utilized to estimate and compare the metabolic coverage of a wide range of molecules with varying hydrophobicity and polarity. In addition to single-phase extractions, we also examined a mix of the polar and apolar phases of the LLE, aiming at increasing the metabolic coverage. Following untargeted RPLC-MS analysis, data were pre-processed using XCMS, MS-FLO and in-house tools, followed by strict rules of feature removal, to omit isotopes, adducts, by-products, background peaks (in procedure blank per method), and also unreliable retention areas (which restricted the inclusion of apolar and larger lipids). The different steps of the pre-processing and the feature yield per step and extraction method are summarized in Table S6. Limited feature annotation was conducted, to assess coverage similarities and differences between the extraction methods. Curation of features, their assessment and metabolite annotation are an ongoing effort as part of building our in-house database of metabolites per biospecimen, species and health condition.

A PCA plot provides an initial impression of the untargeted metabolomics pre-processed results (Figure 4). Apart from fine repeatability of replicate measurements in each extraction method, the PCA illustrates profound differences between the obtained metabolic profiles. PC1 (32%) reflects the polarity of the extraction solutions, separating water-containing solutions from those with higher organic solvent content (right to left, decreasing polarity index). PC2 (27%) is more affected by hydrophobicity and miscibility of the solutions, clearly isolating the LLE-chloroform phase from all others, and not surprisingly closest to the LLE-MTBE extraction. Hierarchical analysis of the untargeted metabolomics results also illustrates the similarities and clustering between the various extraction methods and replicates (Figure S2).

**Figure 5:**
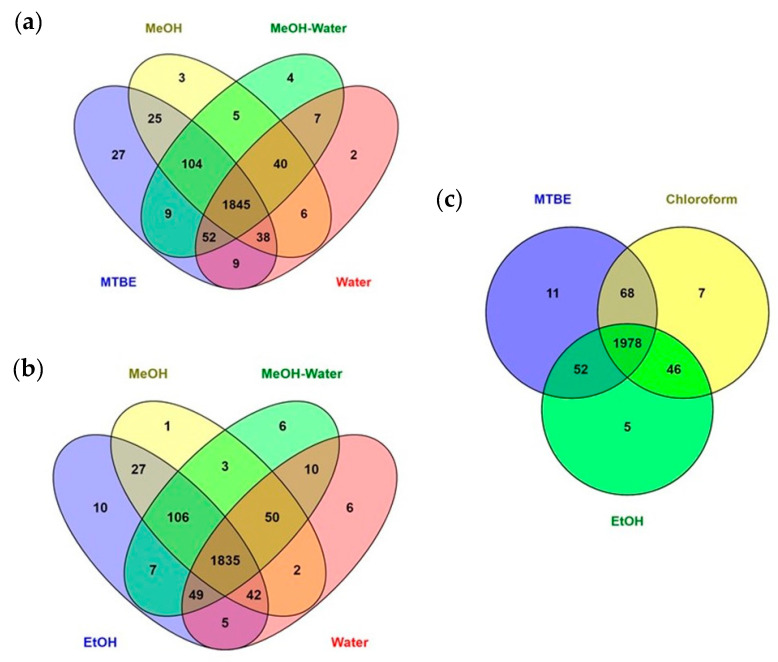
Comparison of the mean numbers of features (triplicate of each extraction protocol); (**a**) comparison of single-phase extractions; (**b**) comparison of MTBE with single-phase extractions; (**c**) comparison of ethanol (which gave the highest number of features among the single-phase extractions) with liquid–liquid extractions.

Adding up all features in the different extracts, a total of 2176 features were detected and retained after data pre-processing, among which the highest coverage was observed for MTBE, chloroform and EtOH extractions (97%, 96% and 91% of total, respectively). Figure 5 summarizes the metabolic coverage overlap in Venn diagrams. Although MTBE and chloroform extractions represented 94% similarity in features, it is clear that MTBE extraction contributes the highest number of unique features, also compared to the more polar extracts. Moreover, MTBE extraction offers an advantage since the protein and cell debris layer is forced to the bottom of the extraction tube following centrifugation, simplifying the removal of both solvent phases. Within the single-phase extraction methods (polar extraction), EtOH provided the highest coverage, probably due to its intermediate polarity.

To demonstrate the effect of the polarity and hydrophobicity of the different solutions on the efficiency of extraction, we present a few examples of standard-identified and putatively annotated features (level 5 identification, with supporting adducts, see Table S7 in Supplementary Excel file). The dominant feature of a metabolite in each extract was selected, if not identical between extracts (for example, the formate adduct of deoxycholate acid was higher only in aqueous extract, while [M-H] was the highest in all other extracts). A putative lignan (enterolactone, eluting at 5 min) recorded the highest peaks in MTBE and in chloroform extracts, followed by the increasing polarity: EtOH, MeOH, MeOH:water, water. A similar pattern was observed for a steroidal hormone (androsterone; rt = 5.5 min) which gave the highest peak in MTBE extract, followed by a 50% decrease in chloroform extract, and a further 100-fold decrease in EtOH and MeOH extracts, and with negligible peaks in MeOH:water and water extracts. Bile acids (rt = 5–6.7 min) were mostly higher in MTBE, lower in MeOH:water extract, and the lowest in water extracts. Variation in residues affected the performance of the chloroform, ethanol and methanol extracts to different degrees. A small polar lipid (e.g., LPC 12:0, rt = 6.5 min), showed the highest peak intensity in EtOH extract, followed by MeOH, then 2–3 fold lower peak intensity in chloroform and MTBE extracts, and 100–1000 fold lower peaks in MeOH-water and water extracts, respectively. More extremely, a putative triterpenoid (rt = 6.8 min) showed the highest peak area in the EtOH extract, a 5-fold decrease in the MeOH extract, and 100–1000 fold decrease in all other extraction methods. Bilirubin degradation products (urobilinogen etc.) showed the lowest peak area in chloroform extracts. Aromatic amino acids (Trp, Tyr, Phe; rt = 2.1–2.8 min) showed the highest peak area in the MTBE extract, and the lowest (5–20 fold decrease) in the chloroform extract. This also tended to be the picture for purines (uric acid, xanthine, inosine and others; rt 1.4–2.6 min), although the later eluters of the class (methyl- and dimethyl-urates) tended to show similar peak areas for all extraction methods. No dramatic differences between extraction methods were observed for polar benzenoids (such as phenylacetic acid and phenol glucuronide). The examples above highlight that no single extraction method provides the best sensitivity for all chemical classes, and it can be beneficial to tailor the method to the priorities of the study (lipids; natural products; various gut-bacteria metabolites etc.).

To further support the reliability of the untargeted measurements, Figure S3 presents the relative peak area of metabolites from different chemical classes (identity confirmed with authentic chemical standards). The same metabolites were analyzed by targeted methods to examine their recovery efficiencies, as discussed in the following section.

### 2.4. Extraction Repeatability and Recovery for Selected Methods

Since MTBE, EtOH and chloroform yielded the highest metabolite coverage; extraction repeatability and recovery were evaluated for these methods only.

Within-day repeatability of the extraction methods was calculated based on the peak areas of 15 spiked stable-isotope labeled standards (Table 1). Overall, the CV values of the MTBE method (CV range 3–28.5%) were lower than those for the chloroform (CV range 1.5–42%) and EtOH (CV range 2–41%) methods. The overlaid total ion chromatogram and extracted ion chromatograms of three different MTBE extractions are illustrated in Supplementary Figure S4.

Extraction recoveries were calculated by the ratio of the stable-isotope labeled standard in the pre-extraction samples to the same-labeled standard in the post-extraction species. Similar to the trend observed for extraction efficiency of endogenous metabolites, MTBE outperformed other methods in extraction recovery. As illustrated in Supplementary Figure S5, D4-DCA, long chain fatty acids, U 13C6-lysine, D6-ornithine, D3-9-15N-aspartate and D2-glycine demonstrated high recovery (≥80%) in the chloroform and MTBE methods. D4- Succinate showed high recovery (70%) in MTBE method only. Overall, EtOH extraction showed a lower recovery for all tested compounds. This clearly indicates the importance of performing recovery tests prior to selecting a solvent for extraction.

The higher CV value and lower recovery in the chloroform method might be attributed to the presence of the interphase (cell debris and protein) between the aqueous and organic layer.

## 3. Discussion

The major objective of this study was to find an optimal and robust sample preparation method for human feces directed to both targeted and untargeted metabolomics analysis, through the application of an LC-MS-driven metabolomics workflow.

### 3.1. Quantity of Starting Material

An important factor that affects the extraction efficiency and recovery is the quantity of starting material; especially for a complex matrix-like feces. Unlike other biofluids, feces is not homogenous and contains different topographical locations. Recently, it has become apparent that the sampling region of the human fecal scoop significantly impacts metabolic profiles [14]. In this regard, many studies solely performed homogenization prior to aliquoting [25,26], and the effect of fecal scoop size on the metabolite composition (i.e., what scoop size can be representative of the whole) has not yet led to a consensus. Gratton et al. [27] recommended 15 g of fecal sample as a representative amount. However, this amount was used for fecal water extraction and the sample size might be too large to be applicable for all cohort studies. Moreover, collected fecal samples from patients are often too large to be easily handled for homogenization. Our data revealed that, in case of homogenization with simple stirring, at least 0.50 g of wet feces from the whole feces scoop is needed as starting material to accurately represent the whole; smaller sample sizes (≤0.50 g) suffered from relatively higher deviations in feature intensity. This might be attributed to the presence of fecal particulates and undigested material such as fibers in the feces. In addition, the effect of the sample type (e.g., diarrhea or constipated) on the required sample size needs to be explored.

### 3.2. Assessment of Extraction Method, Solvent and pH for Optimal Extraction Efficiency

Fecal samples are typically prepared with non-selective sample pretreatment strategies to allow maximum coverage. While extraction with one solvent is common [28], there is an inconsistency between many studies with regard to pH and solvent selection, and the resulting metabolic coverage and extraction efficiency. Deda et al. [29], selected acidic or neutral acetonitrile as solvents with satisfactory extraction coverage for different classes of compounds in rat feces, while neutral propanol provided a better selectivity for compounds such as ornithine, lysine, hypoxanthine and tryptamine. Bascon et al. [30] used MeOH or MeOH followed by ethyl acetate for extraction of polar and apolar metabolites from pig feces. In the study by Turroni et al. [31], who applied a targeted metabolomics approach to profile the human fecal metabolome, MeOH provided a higher yield for metabolites such as sphingomyelin, phospholipids and acylcarnitines, while for amino acids and hexose sugars, a mixture of MeOH and phosphate buffer resulted in a higher yield. Complementary to previous studies, our data revealed that there is no ideal pH value that suits the full range of metabolites with different chemical properties, and obtaining satisfactory extraction efficiency for all metabolites with single-phase extraction is unfeasible.

The potential of LLE for efficient extraction of a broad range of polar and apolar metabolites from different matrices became the focus of recent metabolomics studies. In this regard, the superiority of LLE extraction with MTBE was shown by Sostare et al. [32], who reported a higher reproducibility and efficiency in human plasma and urine. In another study, Patterson et al. [33] demonstrated the strength of the Matyash method (based on MTBE) as compared to the Bligh and Dyer method (based on chloroform) for efficient and reproducible extraction of a broad range of polar metabolites and lipids from plasma. Similarly, in the presented experiment, the MTBE method outperformed LLE with chloroform and single-phase extractions in terms of extraction efficiency for polar and apolar metabolites in feces. Notably, solvents such as MTBE and chloroform can disrupt cells by dissolving membrane lipids, hence facilitating the release of intracellular metabolites and increasing metabolites yield. This is particularly important for complex biological matrices such as feces containing different types of cells. It has been shown that commonly used disruption methods such as sonication and bead beating are not sufficient to entirely lyse thick and waxy cell walls, especially for gram positive and fungal cells [34]. Although fecal metabolites of intact bacterial cells do not impact the human health state, fundamental bias between samples can be generated by partial cell lysis during sample pretreatment steps (i.e., freeze-thaw cycles, sonication, and vortexing). Therefore, a selection of solvents for fecal extraction, such as chloroform and MTBE, in combination with shearing forces can improve the comparability between samples. Overall, data must be interpreted with caution, acknowledging the effect of the release of intracellular metabolites on the final extraction yield.

### 3.3. Extraction Coverage

Untargeted metabolomics aims to cover a broad range of metabolites from biological samples, which largely depend on the sample preparation protocols used for metabolite extraction. Our results demonstrated differences in metabolic coverage and sensitivity between the various extraction methods. Liu et al. reported improvements in metabolite coverage of plasma sample by using two-step LLE in comparison to conventional MeOH extraction [35]. Similarly, Whiley et al. [36] reported a drastic improvement in the coverage and reproducibility of plasma metabolites using LLE extraction with MTBE in comparison to MeOH/EtOH (1:1 *v*/*v*). To the best of our knowledge, we showed for the first time that mixing polar and apolar phases of MTBE extraction improves the coverage of fecal metabolites significantly. Although it is expected that water and acetonitrile (9:1 *v*/*v*) as reconstitution solvent reduce the amount of phospholipids in the final extract, we did not assess the matrix effect for each extraction method in this study. Further studies, which take the matrix effect into account, will need to be undertaken.

### 3.4. Extraction Repeatability and Recovery for Selected Methods

Based on the criteria for repeatability (CV ≤ 35%) and recovery (≥80%) of extraction suggested by previous studies [37], our results suggest that good repeatability and recovery was obtained for the MTBE method, which was satisfactory for metabolomics extraction.

### 3.5. Standardization

One aspect in fecal sample preparation, which has not been explicitly mentioned before is the way to report the amounts or volumes that are being used during sample preparation. Usually the solvent volume of the first step is reported as µL/mg feces, and for the solvents in further steps, it has been reported as *v*/*v* ratio [16,29], which complicates calculating the actual used volumes. We propose to report the solvent volume of each step to the initial fecal material (µL/mg _feces_ or µL/mg _dried feces_), as described in the Materials and Methods Section, to make it easier for others to repeat our method.

In conclusion, the MTBE extraction method exhibits several advantages, including superior extraction efficiency for different classes of compounds, high metabolic coverage (using mixed layers), high repeatability and satisfactory recovery. With interest in both polar and apolar metabolites, a robust biphasic extraction method such as MTBE for the complex matrix of feces can greatly reduce experimental time and cost. Importantly, reporting solvent volumes of each step to the initial dried fecal material (µL/mg feces) offers a major step towards standardization, which enables confident assessment of the contributions of gut bacterial metabolites to human health.

Despite some limitations (low number of samples and donors and incomplete chemical representation), this work shows how commonly applied feces sample preparation techniques compare in their performance for targeted and untargeted metabolomics. Additionally, it provides guidance on the optimization and standardization of fecal sample preparation, which is highly valuable in the further exploration of the effect of gut microbiota on human health and disease.

## 4. Materials and Methods

### 4.1. Chemical and Reagents

Analytical grade solvents acetonitrile (MeCN), methanol (MeOH), ethanol (EtOH), chloroform and formic acid were purchased from Biosolve (Biosolve BV, Valkenswaard, The Netherlands), whereas ammonium formate, methyl tert-butyl ether (MTBE), and leucine-enkephalin were purchased from Sigma–Aldrich (Sigma–Aldrich, Burlington, WV, USA). Ultrapure water was obtained from an arium pro UF/VF water purification system with a Sartopore 0.2 μm filter (Sartorius Stedim, Amersfoort, The Netherlands). The majority of standards and deuterated internal standards were purchased from Sigma Aldrich (Sigma-Aldrich, Zwijndrecht, The Netherlands), Avanti (Avanti Polar Lipids, Alabaster, AL, USA), and Fluka (Honeywell, Netherlands). More detailed information regarding the standard suppliers and utilized concentration can be found in the Supplementary Information (Supplementary Table S1).

### 4.2. LCMS Analysis

Untargeted and targeted analysis were performed on an Acquity UHPLC system coupled to a Synapt_G2S mass spectrometer (Waters Chromatography Europe BV, Etten-Leur, The Netherlands), collecting full scan MS data with a 40–1200 Da mass range. Electrospray ionization was applied in negative mode only. A concentration of 0.1 mg/L Leucine-Enkephalin in water/MeOH/formic acid (50/50/0.1 *v*/*v*/*v*) was used as reference for mass measurement with infusion flow rate of 10 µL/min. MS data were collected in centroid mode. Targeted chromatographic separations were carried out on RP (Waters Acquity UPLC T3 column (2.1 × 100 mm, 1.8 μm)) and hydrophilic interaction liquid chromatography (SeQuant^®^ ZIC-cHILIC (2.1 × 100 mm, 3 μm)). Targeted RP (semi polar and apolar metabolites) followed a linear gradient from 0–99.9% mobile phase B (MeOH with 10 mM ammonium formate) over 11.5 min with an injection volume of 4 µL. For HILIC separation (polar metabolites), a linear from 0–100% mobile phase B (10% MeCN in 5 mM ammonium formate) in 14 min with an injection volume of 2.5 µL. Untargeted metabolomics was performed using RP chromatography which followed a linear gradient from 0–99.9% mobile phase B (MeCN with 0.1% formic acid) in 8 min with an injection volume of 4µL. More details of separation conditions and mass spectrometry parameters are mentioned in Supplementary Table S2.

### 4.3. Quantity of Starting Material

The adequacy of starting fecal material for the extraction was investigated for 4 different amounts (0.25 ± 0.02, 0.50 ± 0.02, 1.00 ± 0.02, and 2.00 ± 0.02 g). For each size, duplicates were weighed from the same homogenized sample. After freeze-drying (RVT400 Refrigerated Vapor Trap, Savant, Buckinghamshire, UK), extraction was performed for all different fecal aliquots in parallel using ice-cold MeOH and water (5:1 *v*/*v*) in a ratio of 6:1 µL/mg feces. Every mixture was homogenized by sonication for 10 min in an ultrasonic bath (Ultrasound cleaning baths, USC-TH, VWR, Amsterdam, The Netherlands), followed by vortex mixing on high speed for 10 min. To maximize protein precipitation, samples were stored at −20 °C for 20 min. Thereafter, samples were centrifuged for 20 min (14,700× *g*, 10 °C). Supernatants were filtered through syringe filters PTFE (0.22 µm) before untargeted RP LC-MS analysis. All samples were measured in triplicate (Supplementary Table S2) and the mean intensity of features was used for further analysis.

### 4.4. Assessment of Extraction Method, Solvent and pH for Optimal Extraction Efficiency

To assess the extraction efficiency for chemically diverse metabolites present in the fecal matrix, first, a standard quality control solution (with 53 compounds from 13 different classes) was used to test the performance of targeted analytical platforms. Identity of analytes was determined based on the selectivity of high resolution mass-to-charge and the retention time of the authentic chemical standards. A pooled sample from 3 individual subjects was used. Firstly, each individual’s whole fecal sample (≅7.00 g) was aliquoted (1.00 g) and homogenized using sonication (10 min) and high-speed vortex (10 min); then aliquots of all three samples were mixed and homogenized (sonication: 5 min, high-speed vortex: 10 min, stirring with steel spatula). Next, aliquots of 0.50 g were prepared from the pool sample and randomly assigned to different extraction conditions. For single-phase extraction, four commonly used extraction solvents in metabolomics, namely EtOH, MeOH, MeOH: water (1:1 *v*/*v*), and water were utilized. Each extraction solvent was added in 4:1 µL/mg of feces. To investigate the effect of pH, all above-mentioned solvents were used in acidic (0.1% formic acid), neutral, and basic (0.1% ammonium hydroxide) conditions. Further steps of extraction for sonication, centrifugation and filtration were the same as Section 4.3. Each sample pellet passed 3 rounds of extraction and peak areas of target analytes in each round were used for further interpretation of the extraction efficiency. LLE was performed using a MTBE/MeOH/water solvent system (here termed MTBE method) and a chloroform/MeOH/water solvent system (here termed chloroform method) according to a previous study (32). However, slight modifications were implemented to obtain a better solvent ratio (3.6/2.8/3.5, *v*/*v*/*v*) applicable for both polar and apolar metabolites in complex fecal texture. Briefly, 75% ice cold MeOH (5.4 µL/mg feces of MeOH and 1.8 µL/mg feces of ultrapure water) was added to 0.50 g of freeze-dried sample in a 15 mL Falcon tube (Corning^®^ CentriStar™, VWR International B.V, Amsterdam, The Netherlands). Following homogenization (as in Section 4.2), for the MTBE method, 3 µL/mg feces of MTBE were added and samples were vortex-mixed for 2 min at high speed. Each monophasic supernatant was transferred to a clean tube, after which phase separation was induced by adding 4.2 µL/mg feces of MTBE and 5 µL/mg feces ultrapure water. Samples were vortex-mixed for 1 min, incubated at room temperature for 10 min and centrifuged for 20 min (14,700× *g*, 10 °C). After precipitation of proteins and debris at the bottom of the tube, the polar (lower layer) and non-polar (upper layer) fractions were transferred into different tubes and dried using a nitrogen blow-down evaporator (Liebisch Labortechnik, Bielefeld, Germany). The residues of lower and upper layers were transferred to separate tubes then reconstituted with 2 mL acetonitrile:water (9:1 *v*/*v*) for both targeted reversed phase and HILIC chromatography. The chloroform method followed the MTBE method ratios with the difference of replacing MTBE by chloroform. Due to the higher density of chloroform, the polar fractions stayed at the top and non-polar fraction at the bottom, while protein and debris precipitated between the layers as an intermediate.

For untargeted metabolomics, single-phase extractions were used in neutral pH only, while LLE extraction polar and apolar layers were combined in a tube before being subjected to evaporation and the residue was reconstituted with 2 mL water and acetonitrile (9:1 *v*/*v*).

It is worth mentioning that in order to increase the quenching and minimize the residual enzymatic activity, all solvents for extraction in this study were used ice-cold.

### 4.5. Assessment of Extraction Repeatibility and Recovery

Only MTBE, chloroform and EtOH extraction methods that yielded the highest number of features in untargeted analysis were included in the repeatability and recovery assessment. To determine the extraction repeatability, the coefficient of variation (CV) was calculated for three replicates of the peak areas of 15 stable-isotope labeled standards (Supplementary Table S1) spiked at a concentration of 5 µM (apolar standards) and 10 µM (polar standards) in 0.50 g of fecal sample.

The recovery was calculated according to the formula below:

(1) %recovery =(Area A)/(Area B)×100

where A represents the peak area of a stable-isotope labeled standard spiked before extraction and B represents the peak area of a stable-isotope labeled standard in an extracted matrix sample spiked post-extraction. The results reported for the CV value and recovery were calculated using the mean value obtained across three different extractions tested for each method.

Before comparing the extraction protocols, five blanks and five standard quality control solutions were injected at the start of the analytical sequence to assure protocol reproducibility, controlling the performance of the UHPLC-MS system.

### 4.6. Data Pre-Processing and Software

For targeted peak picking, the LC-MS data were processed with the TargetLynx application in the Masslynx software (Waters, version 4.1). For untargeted analysis, the raw data sets were converted to mzML format using ProteoWizard software version 3.0 [38]; a retention time cut-off at 600 s and peak picking at MS level 1 was applied during the conversion. The converted data were then processed using XCMS (version 3.8.2) following an optimization of parameters (Supplementary Table S5). Adducts and isotopes were grouped into a single feature with the CAMERA package [39]. For recognition and removal of erroneous features in the datasets, MS-FLO (http://msflo.fiehnlab.ucdavis.edu (accessed on 9 May 2021)) was used. The resulting XCMS table was further processed using the steps explained in Table S4, to preserve only peaks eluting before 7 min, with *s/n* > 10, peaks present in at least 2/3 technical replicates per sample, and at least 5-fold higher intensity in biological samples than the blank procedure (per preparation method).

For data processing and visualization, Python (version 2.7.15) and R (version 3.6.0) were utilized. R packages “tidyverse” and “ggplot2” were used to format the data and plot the figures, respectively. To obtain an overview of the metabolomic data, abundance profiles of metabolites were glog transformed and subjected to principal component analysis (PCA) and dendrogram tree clustering (Pearson distance and average linkage) using MetaboAnalyst version 5.0 (https://www.metaboanalyst.ca (accessed on 20 March 2021)). Venn diagrams were drawn via an online website at (http://bioinfogp.cnb.csic.es/tools/venny/index.html (accessed on 15 March 2021)).

## Figures and Tables

**Table 1:** The coefficient of variation (CV%) for peak areas of 15 stable-isotope labeled standards spiked in fecal samples for MTBE, chloroform and ethanol extraction protocols in negative ESI using untargeted LCMS.

Compounds	LLE-MTBE	LLE-Chloroform	EtOH
D4-DCA	20	14	21
D4-CA	3	42	21
FA 20(4)-d8	6.5	10	10
FA 22 (6)-d5	6.3	15	3.5
D5-TUDCA	4.5	5.6	10.8
D4-GDCA	10	28	10
FA18(2) d4	5	19	11.5
LPE (17:1)	28.5	16	41
D3-Leucine	5	18	8
D4-Succinate	8	11	2
U13-C5-valine	4	1.5	3
D6-Ornithine	7.5	7	24
U 13C6- Lysine	6.5	14	17
D3-9-15N-aspartate	12	28	37.5
D2-Glycine	5	8	14

## Data Availability

The data presented in this study are available in Supplementary Materials.

## Supplementary Materials

The following are available online at https://www.mdpi.com/article/10.3390/metabo11060364/s1, Table S1: Standard information, Table S2: LCMS conditions, Table S3: Partial validation information of targeted HILIC and reversed phase analytical platforms, Table S4: Calibration range used in developing targeted HILIC and reversed phase platforms, Table S5: List of parameters used for untargeted data processing with XCMS, Table S6: Process of untargeted feature cleaning, Table S7: Level 5 identification of features in untargeted analysis of fecal sample extracted with different extraction solutions, Figure S1: Comparison of LLE yield (MTBE) with three rounds of single phase extraction using Ethanol, Figure S2: Hierarchical analysis of the various extraction procedures, Figure S3: Example of the peak area between different extraction methods, Figure S4: The overlaid total ion chromatogram and extracted ion chromatograms of three different MTBE extractions on the same fecal sample, Figure S5: Extraction recovery (% of starting material) for internal standards, following extraction with EtOH, chloroform and MTBE.
