# Supplementary material for: Towards Standards for Human Fecal Sample Preparation in Targeted and Untargeted LC-HRMS Studies
Source: Metabolites. 2021 Jun 7;11(6):364. doi: 10.3390/metabo11060364 (PMC8230323; doi:10.3390/metabo11060364)
Supplement: Supplementary file 1 [file metabolites-11-00364-s001.zip › metabolites-1186948-supplementary/Supplementary information/Metabololites Journal_Appendix.pdf]

metabolites

Metabolites Journal

Supplementary material

Towards standards for human fecal sample preparation in targeted and untargeted LC-HRMS studies

Farideh Hosseinkhani [1], Anne-Charlotte Dubbelman[1], Naama Karu [1], , Amy C. Harms[1] and Thomas Hankemeier [1,*]

[1] Division of System Biomedicine and Pharmacology, Leiden Academic Center for Drug Research, Leiden University, Leiden, Netherlands
* Correspondence: hankemeier@lacdr.leidenuniv.nl; Tel.: +31715274226

Supplementary Table S1. Standards information							
**Mix 1-HILIC**				**Mix 2-reversed phase**			
**Compound**	**CHEBI**	**Conc. (uM)**	**Supplier**	**Compound**	**CHEBI**	**Conc. (uM)**	**Supplier**
Aspartic acid	17053	80.0	Sigma-Aldrich	FA (14:0)	140940	50	Merck
Betaine	17750	150	Sigma-Aldrich	FA (16:0)	140943	100	Sigma
Citrulline	16349	50	Sigma-Aldrich	FA (16:1)	140944	25	Fluka
Glutamic acid	16015	200	Sigma-Aldrich	FA (17:0)	140945	4	Sigma-Aldrich
Glutamine	17061	500	Fluka	FA (18:0)	140947	25	Sigma
Gamma-aminobutyric acid	16865	4	Sigma-Aldrich	FA (18:1)	140948	25	Sigma
Leucine	15603	100	Fluka	FA (18:2)	140949	25	Sigma
Isoleucine	17191	100	Fluka	FA (20:0)	140951	20	Sigma-Aldrich
Lysine	18019	400	Fluka	FA (20:2)	140952	10	Sigma-Aldrich
Ornithine	15729	100	Sigma-Aldrich	FA (20:4)	132539	25	Sigma
pipecolic acid	17964	10	Sigma-Aldrich	FA (20:5)	132540	15	Cayman chemicals
Pyroglutamic acid	16010	40	Sigma-Aldrich	FA (22:0)	140958	20	Sigma-Aldrich
Taurine	15891	200	Fluka	FA (22:1)	132541	20	Sigma-Aldrich
Adenosine	16335	0.5	Fluka	FA (22:6)	132544	20	Cayman chemicals
Adenine	16708	0.5	ALFA AESAR	FA (24:0)	155816	20	Sigma-Aldrich
guanine	16235	4	Sigma-Aldrich	UDCA	9907	100	Sigma
Hypoxanthine	17368	100	Sigma-Aldrich	DCA	28834	100	Brunschwig
Inosine	17596	4	Sigma-Aldrich	CA	16359	100	Brunschwig
Uric acid	27226	1000	Sigma-Aldrich	GCDCA	36274	100	Sigma
Uracil	17568	4	Sigma-Aldrich	GDCA	27471	100	Sigma
Lactic acid	422	1500	Acros organics	TCDCA	16525	100	Sigma
Succinic acid	15741	50	Sigma-Aldrich	TDCA	9410	100	Sigma
Pyruvic acid	32816	100	Sigma-Aldrich	TCA	28865	100	Sigma
Pantothenic acid	7916	6	Sigma-Aldrich	SN1-LPC (18:1)	64566	40	Avanti
Butyric acid	30772	2	Sigma-Aldrich	SN1-LPC (18:2)	64549	40	Avanti
5-hydroxyhexanoic acid	131434	6	Sigma-Aldrich	LPE (18:0)	64576	20	Avanti
**LPE(17:1)**	—	5	Sigma-Aldrich	LPE (18:2)	91296	20	Avanti
**DCA-d4**	—	5	Sigma-Aldrich	**D2-Glycine**	—	10	CDN Isotopes
**CA-d4**	—	5	CDN Isotopes	**D3 Leucine**	—	10	CDN Isotopes
**FA 20(4)-d8**	—	5	Sigma-Aldrich	**D4 succinate**	—	10	Cambridge isotope laboratories
**FA 22 (6)-d5**	—	5	Sigma-Aldrich	**U13-C5-valine**	—	10	Cambridge isotope laboratories
**D5-TUDCA**	—	5	Sigma-Aldrich	**D6 Ornithine**	—	10	CDN Isotopes
**D4-GDCA**	—	5	Sigma-Aldrich	**U 13C6- Lysine**	—	10	Cambridge isotope laboratories
**FA18(2) d4**	—	5	Cambridge isotope laboratories	**D3-9-15N-aspartate**	—	10	Cambridge isotope laboratories

metabolites

Supplementary Table S2. LC MS conditions			
**Mode of separation**	**Analytical separation condition**	**Solvents**	**MS parameters**
Reversed phase-untargeted (**method 1**)<br>Water ACQUITY UPLC T3 column (2.1 × 100 mm, 1.8 μm)	0-3.5 min: 0.1 to 30% B, 3.5-8 min: 99.9% B, 8-12 min 99.9% B, 12.1-15 min: 0.1% B<br>Flow rate: 0.4 mL/ min<br>Column temperature: 40°C	A: water with 0.1% formic acid<br>B: MeCN with 0.1% formic acid	Capillary voltage: 1500 V, sample cone voltage:30 V, source temperature:125 °C, desolvation temperature: 300 °C, desolvation gas: 500 L/h
Reversed phase- lipids **(method 2)**<br>Water ACQUITY UPLC T3 column (2.1 × 100 mm, 1.8 μm)	0-7.5 min: 0.1 to 99.9% B, 7.5-11.5 min: 99.9% B, 11.6 -15 min:0.1% B<br>Flow rate: 0.4 mL/ min<br>Column temperature: 45°C	A: 5:95 MeCN: water with10 mM Ammonium formate<br>B: MeOH with 10 mM Ammonium formate	
HILIC-polar targeted **(method 3)**<br>SeQuant® ZIC-cHILIC (2.1 × 100 mm, 3 μm)	0-9.16 min: 0.1 to 75% B, 9.16-14 min 100% B, 14.1-20 min: 0.1% B<br>Flow rate: 0.25 mL/ min<br>Column temperature: 30°C	A: 90% MeCN and 10% water with 5 mM ammonium formate<br>B: 10% MeCN and 90% water with 5 mM ammonium formate	Capillary voltage: 2000 V, sample cone voltage:45 V, source temperature: 80 °C, desolvation temperature: 250°C, desolvation gas: 500 L/h

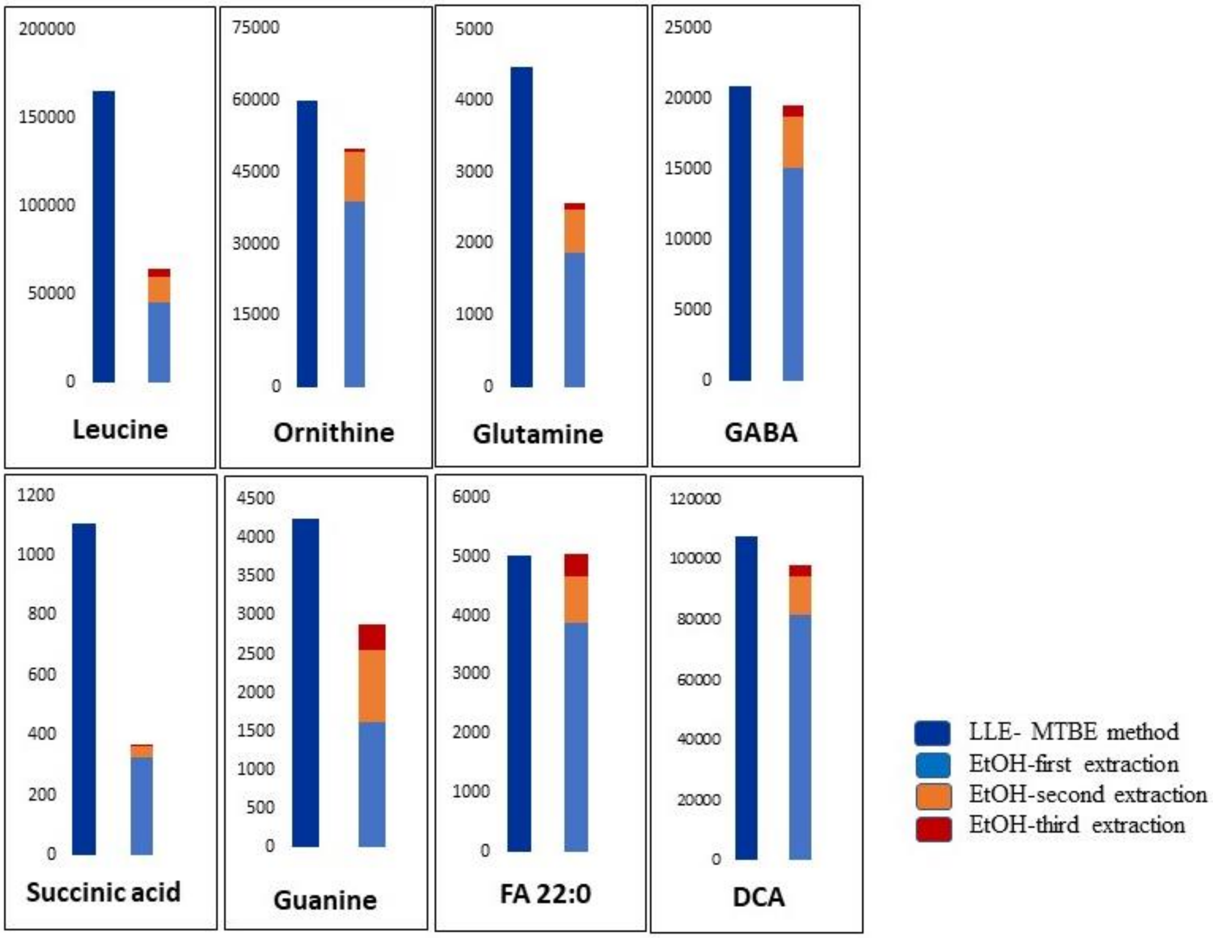

Supplementary Figure S1. Comparison of the LLE metabolic yield between MTBE and three cycles of single-phase ethanol extraction (accumulated bar).

metabolites

Supplementary Table S5. List of parameters used for untargeted data processing with XCMS

**Feature detection**	
Method	CentWave
ppm	5
snthr	10
peakwidth	(3, 8)
mzdiff	0.01
prefilter peaks	3
prefilter intensity	1000
noise	1000
**Retention time correction**	
method	orbiwarp
profStep	1
**Grouping**	
method	density
bw	5
mzwid	0.01
minfrac	0.5
minsample	3
**Annotation**	
feature.Annotation.CAMERA.annotate	isotopes
feature.Annotation.CAMERA.mzabs	0.01
feature.Annotation.CAMERA.ppm	5
feature.Annotation.CAMERA.sigma	6
feature.Annotation.CAMERA.perfwhm	0.6
feature.Annotation.CAMERA.maxcharge	3
feature.Annotation.CAMERA.maxiso	5
feature.Annotation.CAMERA.intensity	Maxo

**Supplementary Table S6**. Process of untargeted feature filtering. The number detailed per extraction in each step denotes the features removed in that step.						
**Cleaning process**	**MTBE**	**Chloroform**	**EtOH**	**MeOH**	**MeOH -Water**	**Water**
Total features detected by XCMS	9830					
Removal of features with rt > 7min	2088	2088	2086	2088	2086	2086
Removal of m/z > 900	718	718	718	718	718	718
Removal of isotopes	1237	1213	1216	1211	1179	1112
removal of non-biological peaks (by blank)	293	448	402	416	550	847
Features with RSD> 35%*	267	217	260	293	271	264
consolidating features per retention time	3208	3047	3067	3038	2960	3604
Final features	2109	2099	2081	2066	2066	1199

*In a case of features with low intensity, no removal based on %RSD was applied

metabolites

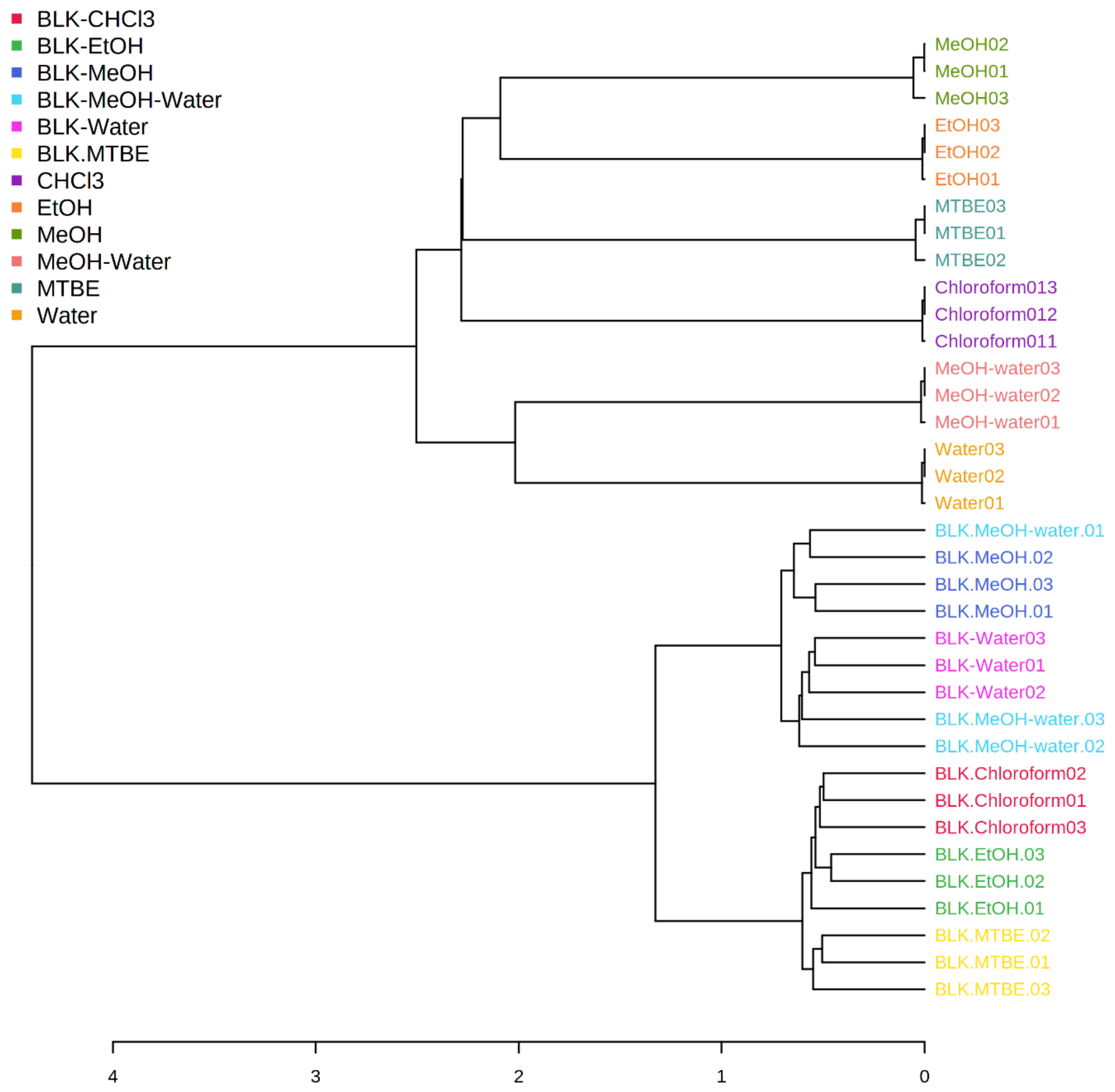

Supplementary Figure S2. Hierarchical analysis of the various extraction procedures, with n=3 replicates each. Clustering was performed based on the Pearson distance and average linkage, on log-transformed and pareto-scaled data. BLK, blank.

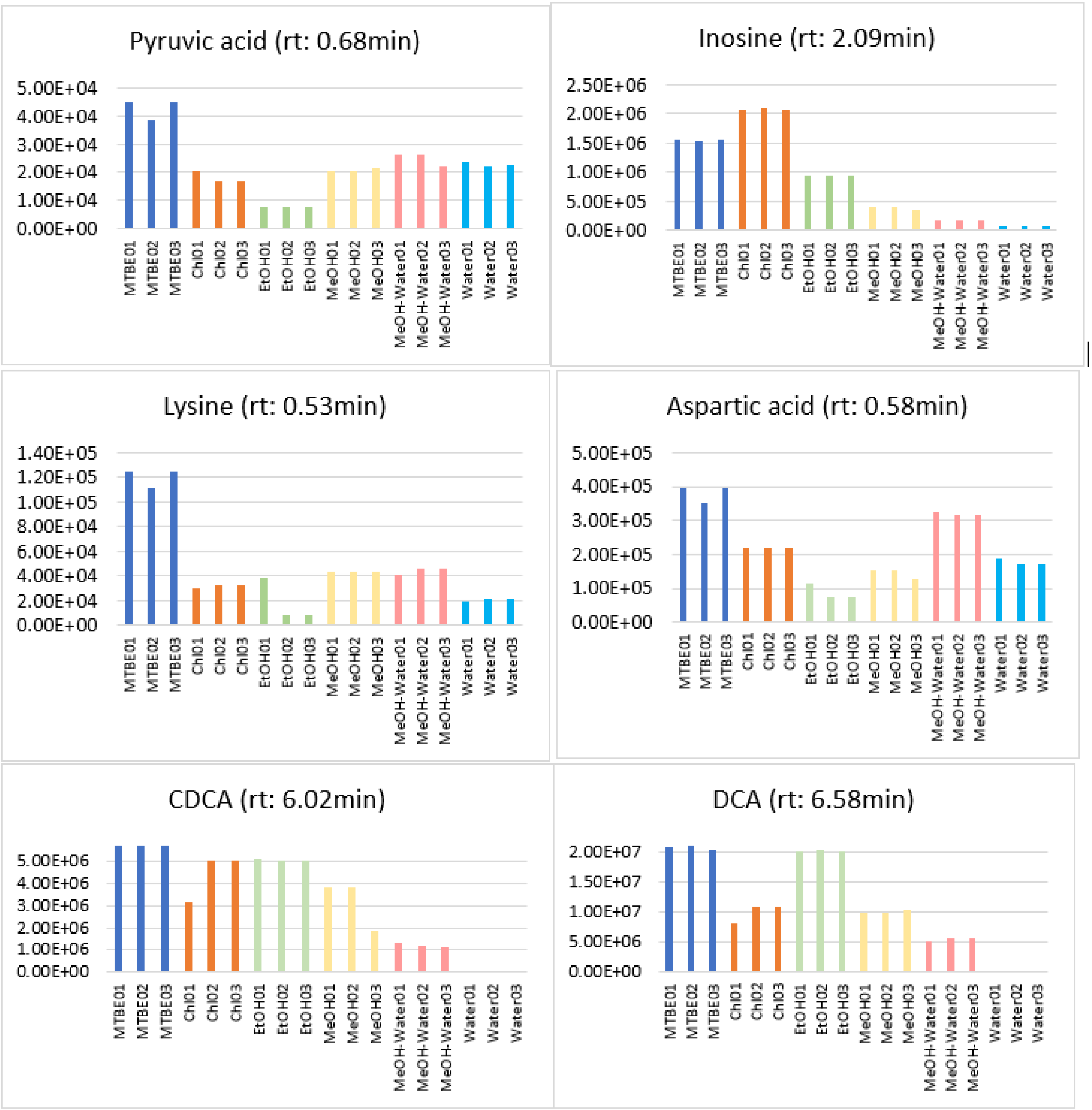

Supplementary Figure S3. Example of the peak area between different extraction methods (LLE versus single phase extraction) in untargeted analysis. Organic solvents exhibit low yield of polar metabolites in the early part of chromatography, and high(er) yield of late eluters.

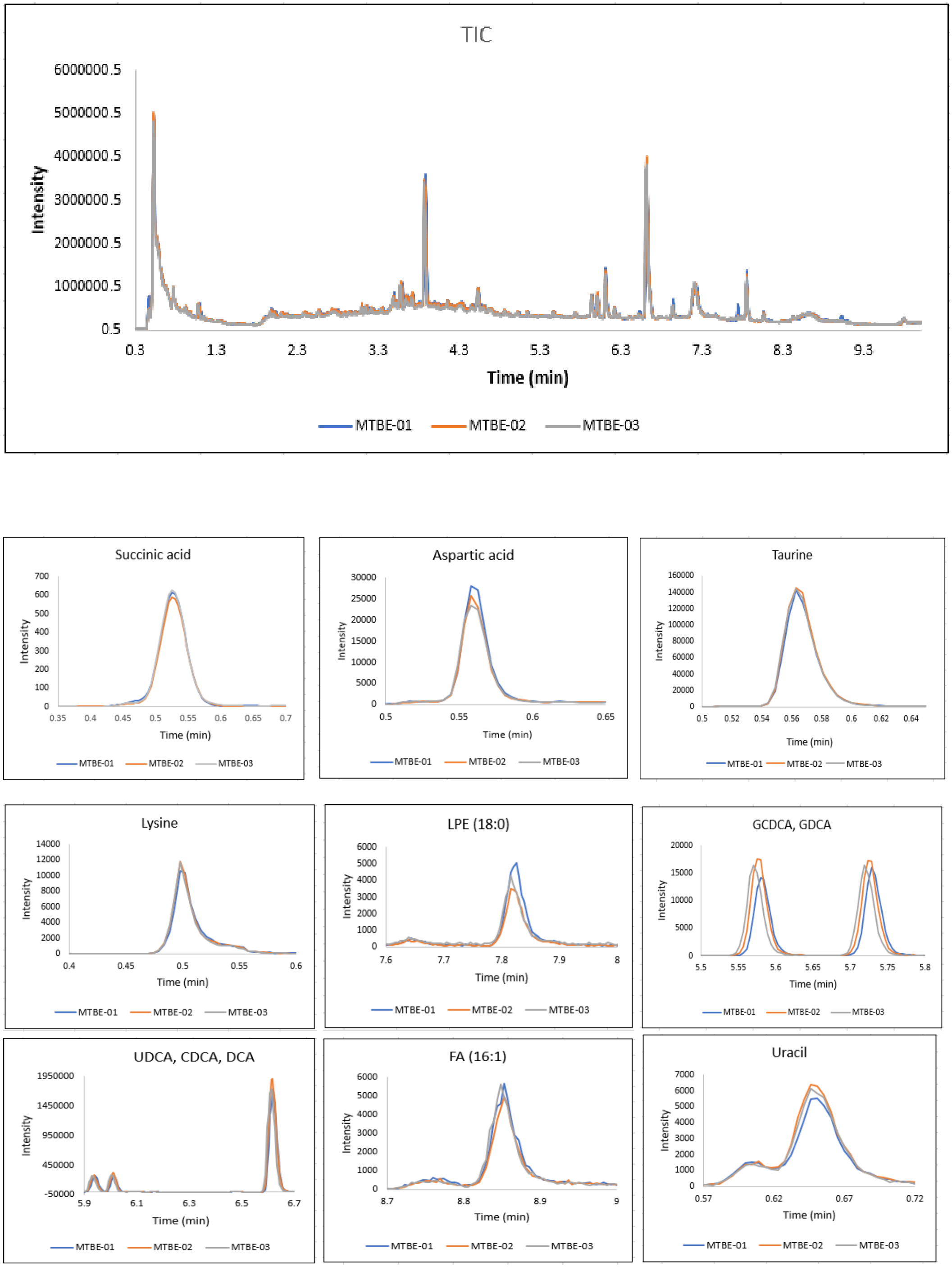

Supplementary Figure S4. The overlaid total ion chromatograms and extracted ion chromatograms of 3 different MTBE extractions on the same fecal sample

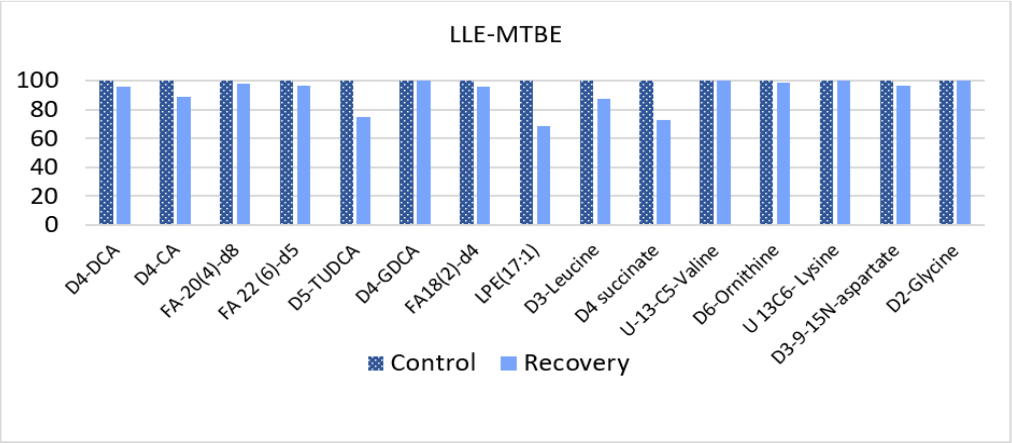

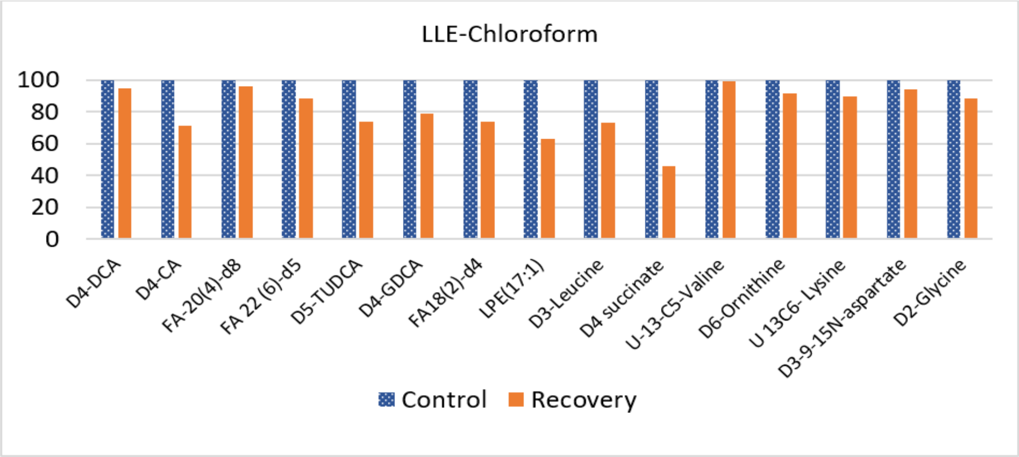

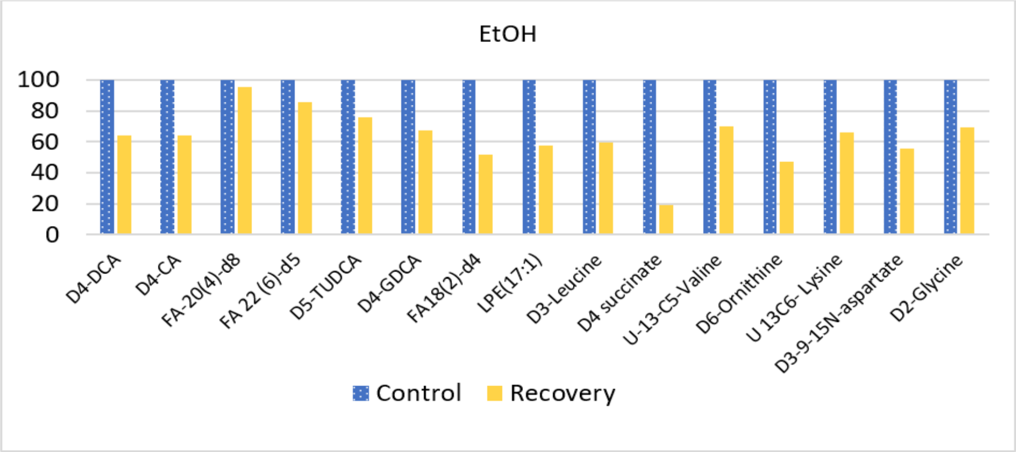

Supplementary Figure S5. Extraction recovery (% of starting material) for internal standards, following extraction with EtOH, chloroform and MTBE. The control sample represents the concentration of internal standard spiked post extraction.
